# Integrated genomic and phenotypic characterization of representative *Salmonella Pullorum* and *Salmonella Enteritidis* isolated from a poultry farm

**DOI:** 10.3389/fmicb.2026.1845729

**Published:** 2026-06-17

**Authors:** Lijun Ding, Jingye Lu, Yalu Zhu, Qiurong Qi, Guiqin Yan, Zefeng Shan, Yu Lu, Hai Xu

**Affiliations:** 1 Jiangsu Agri-animal Husbandry Vocational College, Taizhou, Jiangsu, China; 2 GuoTai (Taizhou) Center of Technology Innovation for Veterinary Biologicals, Taizhou, Jiangsu, China; 3 Yuxi Township Animal Husbandry and Veterinary Station, Taizhou, Jiangsu, China; 4 Institute of Taizhou Agricultural Science, Jiangsu Academy of Agricultural Science, Taizhou, Jiangsu, China

**Keywords:** antimicrobial resistance, poultry, *Salmonella*, virulence, whole-genome sequencing

## Abstract

*Salmonella* remains a major pathogen in poultry production, posing significant threats to animal health and food safety. In this study, two representative isolates, *Salmonella enterica* serovar Enteritidis (SA03) and serovar Pullorum (SA406), were selected from multiple isolates recovered from a diseased poultry farm, as predominant serovars identified in the same production environment, for further characterization. Phenotypic identification, antimicrobial susceptibility testing, whole-genome sequencing, and *in vivo* virulence assessment were performed to provide an integrated genotype–phenotype comparison. Both isolates exhibited typical *Salmonella* characteristics, with SA03 displaying faster growth kinetics than SA406 under tested conditions. Whole-genome sequencing revealed that both isolates harbored a diverse repertoire of antimicrobial resistance (AMR) genes and virulence-associated genes. Notably, SA03 carried a broader range of AMR genes, including *bla*TEM-1B, *aac*(6′)-Iaa, and *sul2*, whereas SA406 exhibited a relatively narrower resistance gene profile. A total of 344 and 340 virulence-associated genes were identified in SA03 and SA406, respectively, with both strains harboring genes related to type III secretion systems and adhesion. Phenotypic antimicrobial susceptibility testing showed partial inconsistencies with genomic predictions. Despite this, *in vivo* virulence was evaluated using specific-pathogen-free (SPF) chickens, revealing LD_50_ values of 10^7.8^ CFU for SA03 and 10^8.5^ CFU for SA406, indicating higher virulence of SA03. This inconsistency suggests that virulence potential may not be directly determined by gene abundance alone. Overall, this study provides an integrated characterization of representative *Salmonella* isolates co-circulating in a poultry farm and highlights the complexity of linking genomic features with phenotypic traits.

## 1 Introduction

*Salmonella enterica* is one of the most important bacterial pathogens affecting poultry worldwide and continues to pose a significant challenge to animal health, food safety, and public health (Jung et al., 2022). Poultry-associated *Salmonella* infections cause a range of clinical outcomes in chickens, from subclinical carriage to systemic disease, resulting in decreased productivity, increased mortality, and persistent contamination of flocks and farm environments (Shaji et al., 2023). Moreover, poultry products remain an important source of human *salmonellosis*, especially outbreaks linked to eggs and poultry meat contaminated with *Salmonella enterica* serovar Enteritidis (*S. enteritidis*) and related serovars, often exhibiting multidrug resistance (MDR) and mobile genetic elements that facilitate dissemination of antimicrobial resistance genes (ARGs) and virulence determinants along the food chain (Perry et al., 2024). In this context, antimicrobial resistance (AMR) in *Salmonella* arises from both intrinsic and acquired mechanisms. While intrinsic resistance is associated with inherent structural or physiological traits, acquired resistance is typically mediated by horizontally transferred genes, which play a major role in the emergence of MDR strains in poultry-associated *Salmonella*. Therefore, *Salmonella* infections in poultry represent a pathogen of dual significance for veterinary health and public health.

Among the numerous *Salmonella* serovars affecting poultry, *Salmonella enterica* serovar Pullorum (*S. Pullorum*) and *S. enteritidis* are of particular concern due to their distinct host interactions and disease outcomes. *S. Pullorum* causes pullorum disease, a systemic infection that primarily affects young chicks and can be transmitted vertically, with chronic or asymptomatic carriage in adult birds, contributing to long-term persistence in flocks (Cui et al., 2024). In contrast, *S. enteritidis* has a broader host range and is commonly associated with subclinical infection in poultry and non-typhoidal salmonellosis in humans (Farhat et al., 2023; Jung et al., 2022). In addition to *S. enteritidis* and *S. Pullorum*, *Salmonella enterica* serovar Typhimurium is also a major serovar associated with poultry and is frequently implicated in foodborne infections. Unlike the host-adapted *S. Pullorum* and the poultry-associated zoonotic pathogen *S. enteritidis*, *S. typhimurium* exhibits a broader host range and is commonly used as a model for studying *Salmonella* pathogenesis (Newson et al., 2022). In this study, the focus on *S. enteritidis* and *S. Pullorum* was based on their co-occurrence as predominant serovars among isolates recovered from the same farm, as well as their distinct epidemiological and pathogenic characteristics. This comparative framework enables investigation of differences between host-adapted and non-host-adapted serovars within the same production environment.

In recent years, whole-genome sequencing (WGS) has become an indispensable tool for high-resolution investigation of *Salmonella* biology, providing comprehensive insights into genomic determinants of virulence, antimicrobial resistance, and population structure (Siddique et al., 2024; Zhou et al., 2025). WGS has been applied to clinical *S. enteritidis* isolates, revealing MDR profiles and the presence of mobile genetic elements such as plasmids and genomic islands that contribute to resistance gene acquisition and propagation (Bu et al., 2024). Similarly, genomic studies of *S. Pullorum* populations from poultry farms have characterized prevalent genotypes and resistance determinants, illustrating the distribution of virulence factors and AMR genes in field isolates (Cheng et al., 2024). Despite these advances, many studies have focused on either phenotypic characterization or genomic analysis alone, and integrated investigations that directly link antimicrobial resistance gene profiles with phenotypic susceptibility testing, as well as virulence gene repertoires with quantitative *in vivo* pathogenicity assessments, remain scarce for co-circulating poultry-associated *Salmonella* serovars.

In this study, two representative isolates belonging to distinct serovars were selected from multiple *Salmonella* strains recovered from the same farm, as they represented predominant and epidemiologically relevant serovars co-circulating within the same production environment. Accordingly, the present study was designed as a focused comparative characterization of representative isolates to investigate their genomic and phenotypic differences, rather than as a comprehensive epidemiological survey. Their growth characteristics were evaluated, and WGS was conducted to determine serovar identity and genomic features through comparative and phylogenetic analyses. We further characterized antimicrobial resistance genes and virulence factors, conducted antimicrobial susceptibility testing using a broad spectrum of antibiotics, and quantitatively assessed virulence by determining the median lethal dose (LD₅₀) in specific-pathogen-free (SPF) chickens. By integrating phenotypic and genomic characterization of field isolates representing both *S. Pullorum* and *S. enteritidis*, this work provides comprehensive insights into the biological properties, antimicrobial resistance patterns, and pathogenic potential of co-circulating *Salmonella* serovars in poultry farms.

## 2 Materials and methods

### 2.1 Isolation and preliminary identification of *Salmonella* isolates

Liver samples were collected aseptically from dead chickens in a diseased poultry farm. The tissues were homogenized under sterile conditions and streaked onto tryptic soy agar (TSA), *Salmonella-Shigella* (SS) agar, and a *Salmonella* chromogenic agar (HB7007-1, Hopebio, Qingdao, China). All plates were incubated aerobically at 37 °C for 18–24 h. Colonies exhibiting typical *Salmonella*-like morphology on the selective media were selected and subsequently purified by subculturing on TSA. Multiple *Salmonella* isolates were initially recovered, from which two representative strains, identified as *Salmonella enterica* serovar Enteritidis (SA03) and serovar Pullorum (SA406), were selected for subsequent in-depth analyses. These two isolates were selected as representative strains of the predominant serovars identified in the farm.

### 2.2 Biochemical identification

Biochemical characterization of isolates SA03 and SA406 was performed using a panel of standard biochemical tests following established protocols commonly applied for the phenotypic identification of *Salmonella* (HKI002, Huankai Microbiological, Guangzhou, China). The tests included peptone water fermentation, urease activity, potassium cyanide (KCN) tolerance with corresponding control medium, lysine decarboxylase activity with decarboxylase control medium, carbohydrate fermentation tests (mannitol, sorbitol, salicin, dulcitol), malonate utilization, and o-nitrophenyl-*β*-D-galactopyranoside (ONPG) reaction. All tests were conducted according to the manufacturer’s instructions and standard microbiological procedures. The reactions were recorded as positive or negative based on color change or turbidity after incubation at 37 °C for the recommended duration.

### 2.3 Growth curve analysis

The growth characteristics of *Salmonella* isolates SA03 and SA406 were evaluated by monitoring bacterial growth at different incubation temperatures. Briefly, overnight cultures of each isolate were inoculated into fresh culture medium and incubated at 37 °C or 42 °C with shaking. The temperature of 42 °C was included to mimic the physiological body temperature of poultry. Following inoculation, bacterial growth was monitored by measuring the optical density at 600 nm (OD₆₀₀) at 1 h intervals using a spectrophotometer. Measurements were performed for a total duration of 8 h, and each time point was assessed in triplicate. The 8 h monitoring period was designed to primarily capture the exponential growth phase, which is most informative for comparative analysis of bacterial growth dynamics. The mean OD₆₀₀ values were calculated and used to generate growth curves for each isolate under the two temperature conditions. The maximum specific growth rate (μmax) was calculated from the slope of the linear regression of log-transformed OD₆₀₀ values during the exponential growth phase (1–3 h). Doubling time was calculated as ln (2)/μmax.

### 2.4 Antimicrobial susceptibility testing

Antimicrobial susceptibility of *Salmonella* isolates SA03 and SA406 was determined using the disk diffusion method in accordance with the Clinical and Laboratory Standards Institute (CLSI) guidelines (CLSI M100-2024, 34th edition). Briefly, bacterial suspensions were prepared from overnight cultures and adjusted to a turbidity equivalent to a 0.5 McFarland standard. The suspensions were evenly spread onto Mueller-Hinton agar plates, and antibiotic disks were applied to the agar surface. A total of more than 20 antimicrobial agents representing different classes were tested, including β-lactams, tetracyclines, fluoroquinolones, aminoglycosides, macrolides, lincosamides, glycopeptides, and folate pathway inhibitors. The plates were incubated aerobically at 37 °C for 18–24 h. The diameters of inhibition zones were measured in millimeters, and the results were interpreted as susceptible (S), intermediate (I), or resistant (R) according to CLSI breakpoints. All tests were performed in duplicate to ensure reproducibility.

### 2.5 Whole-genome sequencing, annotation and visualization

Genomic DNA of *Salmonella* isolates SA03 and SA406 was extracted using the cetyltrimethyl ammonium bromide (CTAB) method with minor modifications. DNA quality and concentration were assessed using a Qubit Fluorometer (Invitrogen, United States) and a NanoDrop spectrophotometer (Thermo Scientific, USA). Whole-genome sequencing was performed using a combination of short-read (Illumina NovaSeq) and long-read sequencing platforms (Nanopore PromethION48 or Pacific Biosciences). Raw reads were quality-filtered to remove adapters and low-quality sequences using AdapterRemoval (Lindgreen, 2012) and SOAPec (Luo et al., 2012). Genome assembly was conducted using SPAdes (Bankevich et al., 2012) and A5-miseq (Coil et al., 2015) for short reads, while Flye (Kolmogorov et al., 2019) and Unicycler (Wick et al., 2017) for long reads, followed by genome polishing with Pilon (Walker et al., 2014). Genome annotation was performed using GeneMarkS v4.32 to predict coding sequences (CDSs). Transfer RNA (tRNA) and ribosomal RNA (rRNA) genes were identified using tRNAscan-SE (Lowe and Eddy, 1997) and Barrnap (version 0.9), respectively. Circular genome maps illustrating genome organization, gene distribution, and GC content were generated using CGView based on the annotated genome assemblies. Genome sequences represent assembled genomes deposited in public databases under the accession numbers provided.

### 2.6 Whole-genome phylogenetic analysis

To infer the evolutionary relationships of *Salmonella* isolates SA03 and SA406, whole-genome-based phylogenetic analysis was performed incorporating representative reference genomes. Representative genomes were selected based on assembly completeness, serovar diversity, and relevance to poultry-associated Salmonella isolates. Complete genome sequences of approximately 30 *Salmonella* strains were downloaded from public databases as references. Genomes in FASTA format were processed with the sketching tool sourmash, which uses MinHash-based k-mer sketching to estimate genome similarity efficiently across large datasets (Irber et al., 2024). Sequence sketches were generated for each genome, and pairwise distances were calculated using the sourmash compare function to produce a distance matrix representing overall genomic similarity (Pierce et al., 2019). A distance-based phylogenetic approach, combining sourmash with the Neighbor-Joining algorithm, was used for rapid genome-wide comparison. Although SNP-based maximum likelihood methods can provide higher resolution, the present approach was considered sufficient for resolving relationships at the serovar level. The distance matrix was exported as a text file and reformatted for phylogenetic analysis. In the R environment, the distance data were imported using the ape package, and a phylogenetic tree was constructed using the Neighbor-Joining algorithm, a distance-based method commonly applied to genome similarity data. Branch support values were mapped onto tree nodes. The final tree was exported in Newick format and visualized for interpretation.

### 2.7 Identification of virulence and antimicrobial resistance genes

Virulence-associated genes were identified by screening the annotated genomes against the Virulence Factors of Pathogenic Bacteria database (VFDB) (Chen et al., 2005). Antimicrobial resistance genes were predicted using the Comprehensive Antibiotic Resistance Database (CARD) (McArthur et al., 2013). Hits with sequence identity and coverage above 80% were considered positive matches for antimicrobial resistance or virulence-associated genes. The numbers and functional categories of virulence and resistance genes were summarized and compared between SA03 and SA406.

### 2.8 Determination of median lethal dose (LD₅₀) in SPF chickens

The virulence of *Salmonella* isolates SA03 and SA406 was evaluated by determining LD₅₀ in SPF chickens. Briefly, bacterial cultures were grown to the exponential phase, harvested, and adjusted to four serial concentrations of 10^8^, 10^9^, 10^10^ and 10^11^ CFU/mL using sterile phosphate-buffered saline (PBS). Each concentration was administered intraperitoneally at a volume of 0.1 mL per chicken. For each dose, 10 SPF chickens were inoculated, resulting in four experimental groups per isolate. Following inoculation, the chickens were monitored daily for a period of 14 days, and mortality was recorded for each group. Chickens were maintained and euthanized as per the protocol, approved by the Institutional Animal Care and Use Committee (IACUC) of the Jiangsu Academy of Agriculture Sciences (SYXK2024-2025, 4 May 2025). All experiments were conducted in accordance with the relevant guidelines and regulations of IACUC and the Institutional Biosafety Committee at the Jiangsu Academy of Agriculture Sciences. The LD₅₀ values of SA03 and SA406 were calculated using the Reed-Muench method based on cumulative mortality data (Reed and Muench, 1938).

### 2.9 Statistical analysis

Data were expressed as mean ± standard deviation (SD) and analyzed using Graph Pad Prism version 9 software. The level of significance was set at (*p* ≤ 0.05).

## 3 Results

### 3.1 Isolation and phenotypic identification of *Salmonella* isolates

**Figure 1:**
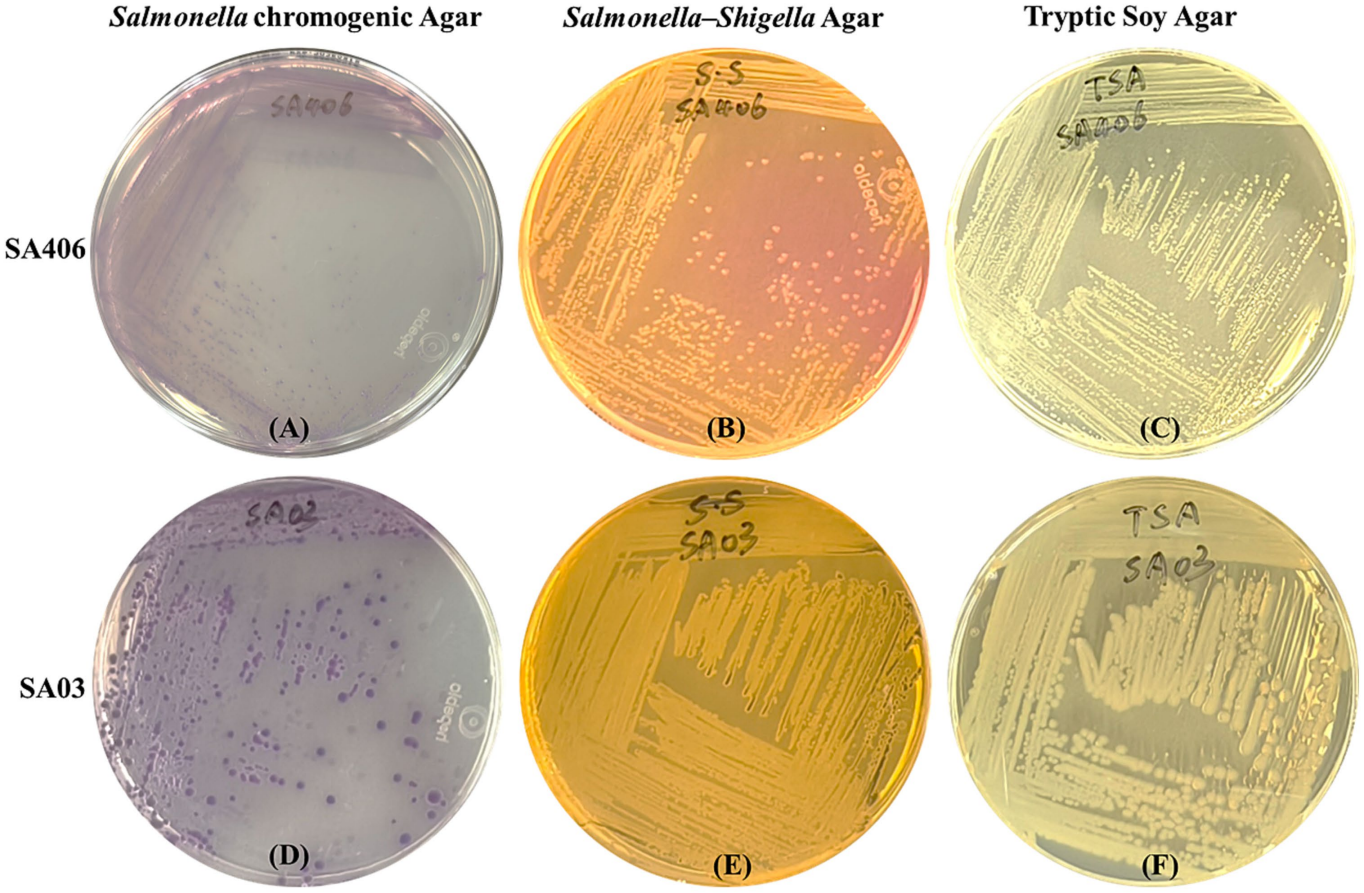
Phenotypic characterization of *Salmonella* isolates SA03 and SA406. **(A–C)** Colony morphology of SA03 on *Salmonella* chromogenic agar, *Salmonella-Shigella* (SS) agar, and tryptic soy agar (TSA), respectively. **(D–F)** Colony morphology of SA406 on *Salmonella* chromogenic agar, SS agar, and TSA, respectively.

Among multiple *Salmonella* isolates recovered from liver samples of diseased chickens on the same farm, two representative strains belonging to different serovars were selected for detailed characterization and designated as SA03 and SA406. When cultured on tryptic soy agar (TSA), *Salmonella-Shigella* (SS) agar, and *Salmonella* chromogenic agar, both isolates produced colony morphologies characteristic of *Salmonella*. On TSA, colonies of SA03 were slightly larger than those of SA406 after identical incubation periods, while both isolates exhibited smooth, circular, and moist colonies. On SS agar, pale colonies with or without black centers were observed, consistent with hydrogen sulfide production. On *Salmonella* chromogenic agar, both isolates formed colonies displaying typical *Salmonella*-associated coloration (Figure 1).

Biochemical characterization results were consistent with the typical profiles of *Salmonella*, providing presumptive identification of the isolates (Table 1). Both SA03 and SA406 were negative for urease activity, potassium cyanide (KCN) tolerance, malonate utilization, dulcitol fermentation, and salicin fermentation, and positive for lysine decarboxylation and mannitol fermentation. Differences between the two isolates were observed in carbohydrate utilization patterns: SA03 was positive for sorbitol fermentation and ONPG activity, whereas SA406 was negative for both reactions. These phenotypic and biochemical profiles were consistent with *Salmonella* and suggested differences at the serovar level.

**Table 1:** Biochemical tests for phenotypic characterization of *Salmonella.*

Test	Results	
	Strain SA03	Strain SA406
Peptone water	−	−
Urease test	−	−
Potassium cyanide (KCN) test	−	−
Potassium cyanide (KCN) control	+	+
Lysine decarboxylase test	+	+
Decarboxylase control medium	−	−
Mannitol fermentation test	+	+
Sorbitol fermentation test	+	−
Salicin fermentation test	−	−
Malonate utilization test	−	−
Dulcitol fermentation test	−	−
o-Nitrophenyl-β-D-galactopyranoside (ONPG) test	+	−

“+” stand for positive; “-” stands for negative.

### 3.2 Growth characteristics of *Salmonella* isolates under different temperature conditions

**Figure 2:**
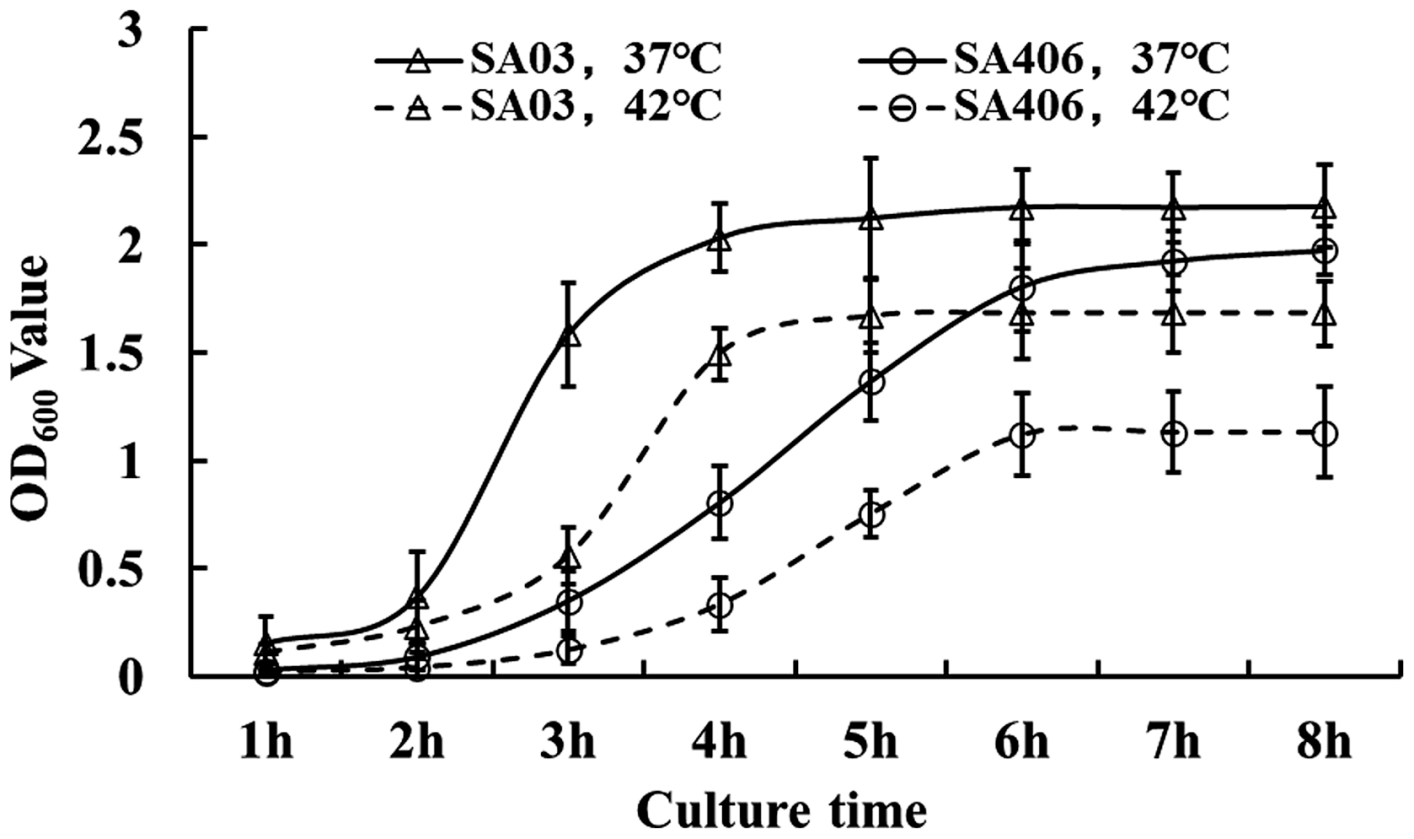
Growth curves of *Salmonella* isolates SA03 and SA406 under different temperature conditions. Growth kinetics of SA03 and SA406 were determined by measuring optical density at regular intervals during incubation at 37 °C and 42 °C. Data represent the mean values of three independent replicates at each time point.

The *in vitro* growth characteristics of SA03 and SA406 were assessed at 37 °C and 42 °C by monitoring optical density over time. Under both temperature conditions, the two isolates exhibited typical bacterial growth patterns, including an initial lag phase followed by exponential growth and a stationary phase (Figure 2). At 37 °C, both isolates showed rapid growth during the early incubation period, with comparable overall growth trends. Growth at 42 °C resulted in altered growth dynamics for both isolates, characterized by a prolonged lag phase and a reduced growth rate relative to 37 °C. Despite this temperature-associated effect, sustained growth was observed for both strains at 42 °C. To quantitatively compare growth dynamics, the maximum specific growth rate (μmax) was calculated based on the exponential phase of the growth curves. At 37 °C, SA03 and SA406 exhibited μmax values of 1.16 h^−1^ and 1.24 h^−1^, respectively. At 42 °C, the growth rates decreased to 0.81 h^−1^ for SA03 and 0.86 h^−1^ for SA406. Correspondingly, the doubling times increased under elevated temperature conditions. These results indicate that SA406 showed a slightly higher growth rate than SA03 under both conditions, while elevated temperature significantly reduced the growth capacity of both isolates.

### 3.3 Whole-genome features and gene family composition of *Salmonella* isolates

**Figure 3:**
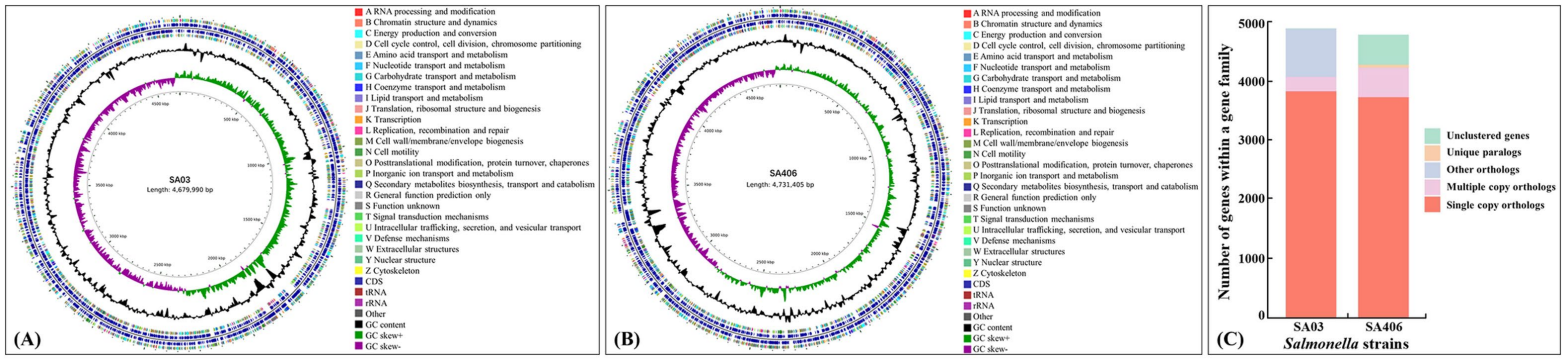
Whole-genome circular maps and gene family comparison of *Salmonella* isolates SA03 and SA406. **(A)** Circular genome map of SA03 showing the distribution of predicted coding sequences, GC content, and GC skew across the chromosome. **(B)** Circular genome map of SA406 showing the distribution of predicted coding sequences, GC content, and GC skew across the chromosome. **(C)** Comparison of gene family composition between SA03 and SA406, showing the number of genes per gene family in each isolate.

Whole-genome sequencing demonstrated that both SA03 and SA406 were assembled into complete circular chromosomes with high assembly quality. The genome of SA03 consisted of a circular chromosome of 4,679,990 bp along with two plasmids (64,327 bp and 29,336 bp), whereas SA406 harbored a chromosome of 4,747,589 bp and a single plasmid of 94,467 bp. The total genome sizes were approximately 4.77 Mb for SA03 and 4.84 Mb for SA406, with comparable GC contents (52.18 and 52.17%, respectively). Both assemblies showed 100% completeness and high sequencing depth (>250×), indicating robust genome quality. Circular genome maps revealed a highly conserved chromosomal organization in both isolates, including the distribution of predicted coding sequences, GC content, and GC skew (Figures 3A,B). Coding sequences were evenly distributed along the chromosomes, and no large-scale structural rearrangements were observed. Despite the overall similarity in genome architecture, minor differences in genomic composition were detected. Local variations in GC content and GC skew were observed in specific genomic regions, suggesting subtle genomic divergence between the two isolates. Comparative analysis of gene family composition further highlighted these differences (Figure 3C). Although most gene families were similarly represented in SA03 and SA406, SA406 exhibited a higher number of gene families with increased gene copy numbers, whereas SA03 showed a relatively more compact gene family profile with fewer multi-copy gene families.

### 3.4 Phylogenetic analysis based on whole-genome comparison

**Figure 4:**
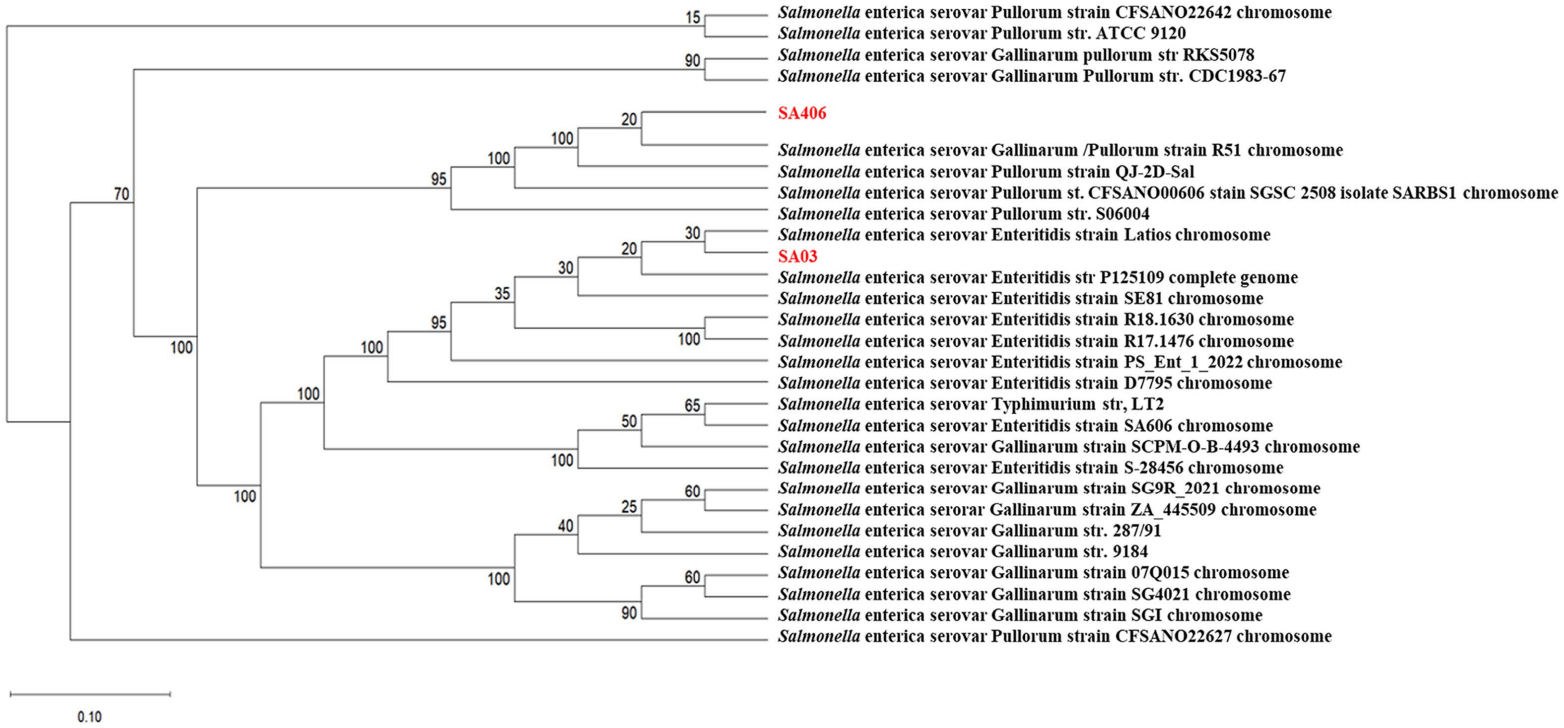
Whole-genome-based phylogenetic analysis of *Salmonella* isolates SA03 and SA406. Phylogenetic tree constructed based on whole-genome sequence alignment of SA03, SA406, and representative *Salmonella* reference strains. The positions of SA03 and SA406 are indicated, showing their clustering with *Salmonella enterica* serovar *Enteritidis* and serovar *Pullorum*, respectively.

Whole genome based phylogenetic analysis was performed to clarify the evolutionary relationships of SA03 and SA406 in comparison with representative *Salmonella* reference strains. The resulting phylogenetic tree showed that the two isolates clustered into distinct lineages corresponding to their genomic identities (Figure 4). SA03 grouped closely with reference strains of *Salmonella enterica* serovar Enteritidis, whereas SA406 clustered with strains of *Salmonella enterica* serovar Pullorum. The two isolates were phylogenetically distant from each other, despite originating from the same diseased chicken farm. These results confirmed the serovar-level assignment of SA03 and SA406 and indicated substantial genomic divergence between the two isolates.

### 3.5 Antimicrobial resistance profiles of *Salmonella* isolates

**Figure 5:**
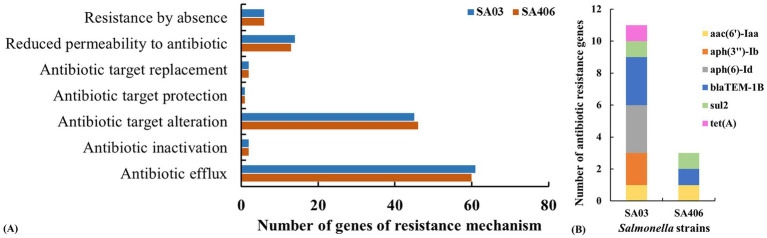
Genomic and phenotypic analysis of antimicrobial resistance in *Salmonella* isolates SA03 and SA406. **(A)** Distribution of antimicrobial resistance genes identified in SA03 and SA406 based on genome analysis. **(B)** Comparison of the total number of antimicrobial resistance genes between SA03 and SA406. Phenotypic antimicrobial susceptibility results corresponding to these genomic findings are summarized in Table 2.

Genome based screening identified multiple antimicrobial resistance associated genes in both SA03 and SA406, with clear differences in gene composition and abundance (Figure 5A). SA03 harbored a broader range of resistance genes, particularly those associated with aminoglycoside resistance, whereas SA406 carried fewer resistance determinants. Quantitative comparison further demonstrated that the total number of resistance genes was higher in SA03 than in SA406 (Figure 5B).

Phenotypic antimicrobial susceptibility testing was conducted to assess whether the genomic resistance profiles corresponded to antibiotic resistance patterns (Table 2). Both isolates exhibited resistance to several β-lactam antibiotics, including penicillin, oxacillin, and amoxicillin, consistent with the presence of β-lactam resistance-associated genes. Differences in susceptibility to tetracyclines were observed between the two isolates: SA03 was resistant to tetracycline, minocycline, and doxycycline, whereas SA406 remained susceptible. Both isolates were generally susceptible to most aminoglycosides tested, despite the detection of aminoglycoside resistance–associated genes in the genome.

**Table 2:** Results of drug sensitivity test for *Salmonella.*

Class	Antibiotics	Disc content (ug)	Judgement standard (mm)			Strain SA03		Strain SA406	
			R	I	S	IZD (mm)	Result	IZD (mm)	Result
β-lactams	Penicillin	10	≤28	-	≥29	0	R	0	R
β-lactams	Oxacillin	1	≤10	11–12	≥13	0	R	0	R
β-lactams	Amoxicillin	25	≤13	14–16	≥17	0	R	0	R
β-lactams	Mezlocillin	75	≤14	15–17	≥18	10	R	0	R
Tetracyclines	Tetracycline	30	≤11	12–14	≥15	0	R	20	S
Tetracyclines	Minocycline	30	≤12	13–15	≥16	12	R	20	S
Tetracyclines	Doxycycline	30	≤12	13–15	≥16	8	R	20	S
Fluoroquinolones	Ofloxacin	5	≤12	13–15	≥16	20	S	18	S
Fluoroquinolones	Enrofloxacin	10	≤16	17–20	≥21	18	I	20	I
Fluoroquinolones	Ciprofloxacin	5	≤15	16–20	≥21	25	S	16	I
Fluoroquinolones	Norfloxacin	10	≤12	13–16	≥17	20	S	13	I
Fluoroquinolones	Levofloxacin	5	≤13	14–16	≥17	20	S	13	R
Aminoglycosides	Gentamicin	120	≤12	13–14	≥15	20	S	20	S
Aminoglycosides	Kanamycin	30	≤13	14–17	≥18	18	S	17	I
Aminoglycosides	Tobramycin	10	≤12	13–14	≥15	15	S	13	I
Aminoglycosides	Amikacin	30	≤14	15–16	≥17	20	S	20	S
Cephalosporins	Cephalexin	30	≤14	15–17	≥18	15	I	0	R
Cephalosporins	Ceftazidime	30	≤17	18–20	≥21	17	R	23	S
Cephalosporins	Ceftriaxone	30	≤19	20–22	≥23	22	I	28	S
Macrolides	Erythromycin	15	≤13	14–22	≥23	0	R	0	R
Macrolides	Azithromycin	15	≤13	14–17	≥18	20	S	14	I
Lincosamides	Lincomycin	2	≤15	16–20	≥21	0	R	0	R
Lincosamides	Clindamycin	2	≤14	15–20	≥21	0	R	0	R
Sulfonamides	Trimethoprim	5	≤10	11–15	≥16	23	S	30	S
Sulfonamides	Sulfamethoxazole	300	≤10	11–15	≥16	0	R	0	R
Glycopeptides	Vancomycin	30	≤14	15–16	≥17	0	R	0	R

The results were interpreted according to the Clinical and Laboratory Standards Institute (CLSI) guidelines (CLSI M100, latest edition). “R” stands for resistant; “I” stands for intermediary; “S” stands for sensitive. “IZD” stands for inhibition zone diameter.

In combination, phenotypic resistance patterns were largely consistent with genomic predictions, although partial discrepancies between genotype and phenotype were observed.

### 3.6 Virulence-associated gene profiles and *in vivo* pathogenicity of *Salmonella* isolates

**Figure 6:**
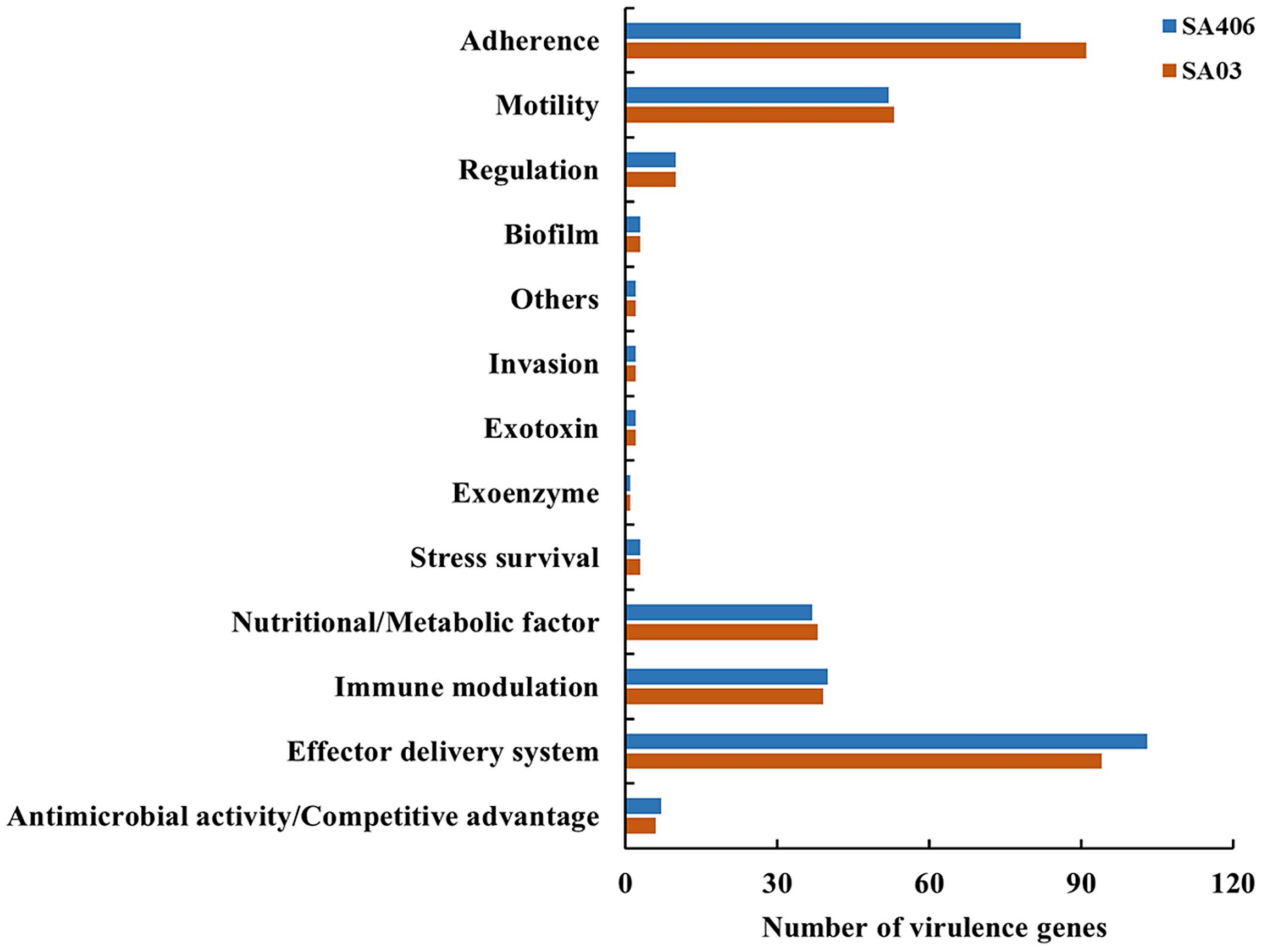
Distribution of virulence-associated genes in *Salmonella* isolates SA03 and SA406. Virulence-associated genes were identified based on genome-wide analysis and categorized according to functional classes. The number of virulence genes in each category is shown for SA03 and SA406.

Genome wide analysis revealed that both SA03 and SA406 carried a broad array of virulence-associated genes related to adhesion, invasion, intracellular survival, and secretion systems (Figure 6). The overall virulence gene profiles of the two isolates were largely similar; however, SA406 harbored a slightly higher number of virulence-associated genes than SA03. To assess pathogenicity *in vivo*, LD₅₀ were determined in SPF chickens following intraperitoneal inoculation (Table 3). The LD₅₀ of SA03 was calculated as 10^7.8^ CFU/mL, whereas that of SA406 was 10^8.5^ CFU/mL. These results indicate that both isolates were pathogenic to chickens, with SA03 exhibiting higher virulence under the experimental conditions.

**Table 3:** Median lethal dose (LD₅₀) of *Salmonella* isolates.

Strain	Animal model*	Route	LD_50_ (CFU/mL)	Calculation method
SA03	SPF chicken	Intraperitoneal injection	10^7.8^	Reed-Muench
SA406	SPF chicken	Intraperitoneal injection	10^8.5^	Reed-Muench

* Specific pathogen-free White Leghorn chickens were used in this study.

Taken together, genomic analysis and animal infection experiments demonstrated that both isolates possessed substantial virulence potential. Notably, the isolate carrying a higher number of virulence-associated genes did not exhibit a lower LD₅₀, suggesting that virulence gene abundance alone may not directly predict in vivo pathogenicity.

## 4 Discussion

*Salmonella* remains a critical threat to poultry production and public health due to their capacity for widespread dissemination, AMR, and pathogenicity. In the present study, we integrated phenotypic characterization, WGS, antimicrobial susceptibility testing, and *in vivo* virulence assessment to compare two *Salmonella* isolates from a diseased chicken farm. This multi-layered approach allowed us to delineate both conserved and divergent biological features of the isolates, reflecting the complex nature of *Salmonella* epidemiology in poultry settings.

In the present study, several resistance determinants identified in SA03 and SA406 are consistent with acquired resistance, including genes associated with β-lactam and aminoglycoside resistance, which are commonly reported to be plasmid-borne. These elements may facilitate the dissemination of resistance traits within bacterial populations. Meanwhile, discrepancies between genotypic predictions and phenotypic susceptibility may also reflect the contribution of intrinsic resistance mechanisms, as well as factors such as gene expression levels or regulatory control. Together, these findings highlight the complexity of antimicrobial resistance and underscore the importance of integrating genomic context with phenotypic data. For example, genomic analyses of *S. Pullorum* isolates from poultry in China similarly demonstrated a high prevalence of AMR genes such as *aac* (6′) -Iaa, *bla*TEM-1B, and *sul2*, underscoring their contribution to resistant phenotypes in field strains (Cheng et al., 2024). In addition, comparable studies on *Salmonella* isolates from poultry sources have revealed substantial carriage of virulence-associated genes related to type III secretion systems and adhesion factors, which were associated with pathogenic potential in both *Pullorum* and non-typhoidal serovars (Lozano-Villegas et al., 2023). In recent years, *Salmonella* strains in chicken and other poultry products in Asian counties have become increasingly resistant to most of the commonly used antibiotics, such as fluoroquinolones, β-lactams, tetracyclines, and aminoglycosides (Chu et al., 2024). Our genomic and phenotypic data showed that SA03 harbored a broader repertoire of AMR genes and exhibited a more complex resistance gene profile than SA406, particularly for aminoglycoside and β-lactam classes. This is aligned with findings from studies of poultry-associated *Salmonella* where multidrug resistance is commonly observed and linked to specific resistance gene combinations, including *bla* and aminoglycoside modifiers that co-occur with mutational mechanisms (Sia et al., 2021).

However, we also observed discrepancies between the presence of resistance genes and phenotypic susceptibility for certain agents, reflecting the notion that genotypic predictions do not always translate into phenotypic resistance. Such inconsistencies may be attributed to several factors, including the presence of incomplete or non-functional resistance genes, as well as low gene expression under *in vitro* conditions. In addition, the genomic context of resistance determinants, such as their localization on plasmids or chromosomes and their association with mobile genetic elements, may influence their phenotypic expression. These findings underscore the importance of integrating genomic and phenotypic data when evaluating antimicrobial resistance.

In terms of virulence potential, although SA406 carried a slightly higher number of virulence-associated genes, SA03 exhibited a lower LD₅₀ value, indicating higher virulence *in vivo*. This discrepancy suggests that virulence potential may not be directly correlated with gene abundance alone. Possible explanations include differences in gene expression levels, the presence of pseudogenes or mutations affecting gene functionality, and variations in regulatory networks controlling virulence gene activation. In addition, the integrity and activity of *Salmonella* pathogenicity islands, which play critical roles in host invasion and intracellular survival, may contribute more significantly to pathogenicity than the total number of virulence genes. This observation underscores an important concept emerging from genomic epidemiology: virulence gene abundance does not necessarily correlate directly with *in vivo* pathogenicity (Shestov et al., 2015). Similar conclusions have been reported in broader comparative studies of *Salmonella*, showing that the presence of canonical virulence factors does not uniformly predict disease severity across serovars or host contexts, as pathogenic outcomes may depend on differential contributions of *Salmonella* pathogenicity island (SPI) loci and variations in effector protein repertoires (Nazari Moghadam et al., 2023). These findings highlight the limitation of inferring pathogenic potential solely from genomic inventories and emphasize the necessity of functional validation.

Phylogenetically, SA03 clustered with reference strains of *S. enteritidis* and SA406 with *S. Pullorum*, reflecting serovar-specific evolutionary backgrounds. This pattern mirrors the broader epidemiological distribution of *Salmonella* serovars in poultry and their host associations. Notably, previous studies have shown that clonal lineages of *Salmonella* often exhibit conserved virulence and antimicrobial resistance profiles. While such genomic consistency may suggest adaptation to similar environmental or host-associated conditions, it does not by itself provide direct evidence of ecological niche association. For example, core genome multilocus sequence typing analysis (cgMLST) and single nucleotide polymorphism (SNP) analyses of duck-derived *Salmonella* isolates revealed clonally related strains across multiple cities, suggesting clonal spread across regions (Lu et al., 2024). Similar clonal dissemination of ST19 *S. typhimurium* has been documented within a poultry breeding system and linked to human clinical isolates, highlighting cross-host transmission potential (Zhang et al., 2026). These findings underscore the value of WGS for high-resolution strain differentiation and epidemiological tracking.

This study has several limitations that should be acknowledged. Only two isolates were subjected to in-depth analysis, these strains were selected as representative isolates from multiple *Salmonella* strains recovered from the same farm. Therefore, the study design aimed to provide a focused comparison between two epidemiologically relevant serovars co-circulating within a single production environment, rather than to perform population-level analysis. This approach reflects field-relevant conditions, where multiple serovars may co-exist, and comparative analysis of representative isolates can provide insights into their differential pathogenic potential. In addition, functional validation of individual antimicrobial resistance or virulence determinants was not performed, and therefore causal links between specific genetic features and phenotypic traits could not be experimentally confirmed. Nonetheless, the present findings are consistent with a growing body of literature demonstrating substantial antimicrobial resistance gene burdens and diverse virulence gene repertoires among poultry-associated *Salmonella* strains. Importantly, our results further highlight the complexity of translating genomic content into phenotypic outcomes, particularly with respect to antimicrobial susceptibility and *in vivo* pathogenicity. Such integrative genotype–phenotype analyses provide valuable context for surveillance and risk assessment efforts and remain essential for addressing the continuing challenges posed by resistant and virulent *Salmonella* strains to poultry production and food safety worldwide. These findings have practical implications for surveillance programs by enabling more accurate identification of high-risk strains and improving evidence-based intervention strategies in poultry production systems.

## Data Availability

The datasets presented in this study can be found in online repositories. The names of the repository/repositories and accession number(s) can be found at: https://www.ncbi.nlm.nih.gov/genbank/, JBUDAV000000000; https://www.ncbi.nlm.nih.gov/genbank/, JBUEGL000000000.
